# Transforming clinical reasoning—the role of AI in supporting human cognitive limitations

**DOI:** 10.3389/fdgth.2025.1715440

**Published:** 2026-01-05

**Authors:** Colin John Greengrass

**Affiliations:** School of Medicine, Royal College of Surgeons in Ireland – Medical University of Bahrain, Busaiteen, Bahrain

**Keywords:** artificial intelligence, clinical decision support, clinical reasoning (CR), cognitive biases, explainable AI (XAI)

## Abstract

Clinical reasoning is foundational to medical practice, requiring clinicians to synthesise complex information, recognise patterns, and apply causal reasoning to reach accurate diagnoses and guide patient management. However, human cognition is inherently limited by factors such as limitations in working memory capacity, constraints in cognitive load, a general reliance on heuristics; with an inherent vulnerability to biases including anchoring, availability bias, and premature closure. Cognitive fatigue and cognitive overload, particularly apparent in high-pressure environments, further compromise diagnostic accuracy and efficiency. Artificial intelligence (AI) presents a transformative opportunity to overcome these limitations by supplementing and supporting decision-making. With AI's advanced computational capabilities, these systems can analyse large datasets, detect subtle or atypical patterns, and provide accurate evidence-based diagnoses. Furthermore, by leveraging machine learning and probabilistic modelling, AI reduces dependence on incomplete heuristics and potentially mitigates cognitive biases. It also ensures consistent performance, unaffected by fatigue or information overload. These attributes likely make AI an invaluable tool for enhancing the accuracy and efficiency of diagnostic reasoning. Through a narrative review, this article examines the cognitive limitations inherent in diagnostic reasoning and considers how AI can be positioned as a collaborative partner in addressing them. Drawing on the concept of *Mutual Theory of Mind*, the author identifies a set of indicators that should inform the design of future frameworks for human–AI interaction in clinical decision-making. These highlight how AI could dynamically adapt to human reasoning states, reduce bias, and promote more transparent and adaptive diagnostic support in high-stakes clinical environments.

## Introduction

Diagnostic reasoning is foundational to medical practice (1), which through a complex cognitive process, enables clinicians to make sense of patient presentations, synthesise clinical data, arrive at accurate diagnoses and ultimately make management decisions (2).

Traditionally, the process of diagnostic reasoning has been entirely reliant on human cognition, incorporating elements such as pattern recognition, causal reasoning, and context-driven decision-making (3, 4). However, inherent limitations of human cognition can hinder diagnostic reasoning. Human cognition, which, while remarkably adept at many cognitive tasks, is limited by constraints on working memory capacity, temporal decay, susceptibility to cognitive overload and numerous other factors (5–7).

In addition to these inadequacies, a greatly increasing quantity and complexity of clinical data presents growing demands on clinicians (4), which clearly highlights the need to explore potential avenues for supporting and perhaps, augmenting diagnostic reasoning (8).

Artificial intelligence (AI) driven Clinical Decision Support Systems (CDSS) employ machine learning to integrate patient data from imaging, lab results, and clinical documentation, forming diagnostic recommendations (9). These platforms can enhance diagnostic reasoning by facilitating the analysis of complex clinical data, which often exceeds human cognitive limits (10, 11). These are already being employed in radiology and oncology for early disease detection and are already demonstrating considerable capability (12, 13).

AI-driven clinical reasoning systems, notably using large language models (LLMs), have demonstrated considerable capability in complex case management (14). A recent example is that of OpenAI's o1 model, demonstrating very significant improvements, with differential diagnosis generation and quality of diagnostic and management reasoning exceeding human baselines (15). As a result of these findings, healthcare professionals are increasingly recognising the potential of AI to serve as a valuable aid in their workflow (16) and given the presence of readily available models are exploring their utility.

However, despite these advancements, current AI systems do face limitations, including a lack of contextual understanding and the inability to generalise knowledge across different clinical scenarios. This can lead to challenges such as algorithmic bias and a reliance on historical data that may not reflect current patient populations or conditions and which has been shown to lead to skewed diagnostic recommendations (17, 18). Nonetheless, these challenges are not insurmountable. With improved data governance and deliberate efforts to curate more inclusive, demographically diverse training datasets, such limitations may be mitigated (19), suggesting that current concerns could be largely transient as the field matures.

Furthermore, concerns with AI-driven CDSSs regarding transparency have been raised, especially with many deep-learning based systems perceived as “black boxes,” preventing observation of any deeper insights into AI driven decision-making processes (20). However, this issue is currently being addressed with significant development focusing on the introduction of explainable AI (21) into diagnostic tools.

Continued advancements in AI technology, particularly in LLMs and multimodal AI systems, may bring clinical decision support tools closer to human-like adaptability (22). Future AI developments may address current limitations, defined by an ability to learn and adapt, even in real-time, across a wide range of clinical tasks and environments, thus mimicking human cognitive capabilities (23). Notably, more advanced AI systems capable of integrating diverse clinical experiences, adapting to novel situations, and dynamically updating reasoning processes (24), would be able to augment diagnostic reasoning in real-time (25), while being able to cross-reference patient data with clinical guidelines and use probabilistic models to generate insights that complement the clinician's reasoning processes. This will likely become a hugely powerful tool for clinical use.

Through leveraging extensive pretrained and self-trained capabilities across varied real-world contexts, advanced AI systems should be able to process vast amounts of data efficiently, leading to more accurate diagnoses and treatment plans. One promising future direction involves a two-tiered architecture with a clinician-facing front end that uses deterministic methods, such as Bayesian updating (described later), to deliver consistent, evidence-based recommendations, with a back-end module that employs reinforcement learning (RL) to explore and refine alternative diagnostic strategies. This capability is further augmented by the concept of a “hive mind,” where through federated learning, multiple AI instances can share insights from real-time clinical experiences. Using a “big-data” and RL analytical capability (26), this approach allows for the rapid development and dissemination of best practices and insights across a healthcare system, fostering a culture of learning and adaptation. As a result, as AI systems become more integrated into healthcare, the potential for collective artificial intelligence to enhance diagnostic reasoning will likely be a key driver of innovation in the field.

It appears that the future of AI-driven CDSS platforms is rather bright and brimming with opportunity to improve the provision of healthcare. With artificial intelligence-based systems becoming increasingly validated, clinically recognised, and trusted by practitioners, AI is indeed set to occupy increasing significance within the process of diagnostic reasoning (27, 28).

This narrative review explores the intricacies of diagnostic reasoning processes from both human and AI perspectives, with a focus on highlighting the inherent limitations in human cognitive architecture and the potential for AI to mitigate such weaknesses and enhance outcomes. This article does not aim to revisit the extensively documented limitations of AI in diagnostics (29), particularly as advancements in models and broader data representation continue to evolve rapidly, thus any assumptions being likely rapidly outdated.

## Methods—literature search and synthesis

The author included English-language, peer-reviewed reviews, conceptual articles, and rigorously vetted arXiv preprints; excluded were non-English works, conference abstracts, vendor materials, and algorithm-only papers without clinical context. After screening 112 diagnostic and 98 AI records by title/abstract, 87 and 64 full texts were selected for thematic analysis. Through iterative reading cycles, the author identified and grouped key methodological and cognitive constructs into provisional themes, refining these into an integrative framework that aligns AI explainability techniques with strategies for mitigating diagnostic biases and the broader theoretical constructs examined in this review.

### Human cognitive limitations

The limitations of working memory (WM) pose significant challenges for diagnostic reasoning. A fundamental constraint is in working memory's *limited capacity*—restricted to retaining approximately 4–5 discrete items at any given time (5). In diagnostic scenarios that require integrating numerous data points from symptoms, laboratory findings, and patient history, this capacity limitation often leads to cognitive overload.

Working memory also has a transient nature and is subject to *temporal decay,* retaining information for only 10–20 s, unless actively rehearsed (6). This means that current information retained in WM is rapidly replaced with newer information. In a fast-paced clinical environment, this decay necessitates constant effort to maintain and manipulate critical data, thus increasing cognitive load and the likelihood of diagnostic errors. Furthermore, the susceptibility of working memory to *interference* from new or competing information can displace current content (30, 31). For example, interruptions during a patient consultation or the simultaneous presentation of multiple clinical findings can easily disrupt the clinician's reasoning process.

The interaction between working memory and ambiguity in respect to clinical data also plays a role in diagnostic reasoning challenges. When faced with incomplete or conflicting information, with excessive cognitive load, the working memory struggles to maintain coherence, often leading to premature or incorrect conclusions (32, 33).

Working memory plays a crucial role in the cognitive processes involved in distinguishing between semantically similar terms, which can heighten the risk of errors in interpretation (34). This phenomenon is particularly evident in the healthcare sector, where confusion between drug names, whether phonetic or orthographic, can have serious implications for patient safety (35, 36).

Finally, the limitations of working memory in diagnostic reasoning are amplified by its reliance on long-term memory (LTM), which stores information in schemata (singular: schema). Schemata are frameworks stored in long-term memory that form the basis of understanding and interpreting knowledge, concepts, causal relationships, and contextual implications (6, 37). In terms of neurocognitive architecture, schemata are purported to be represented as complex arrays of synaptic connections within cortical networks, which are activated in response to sensory cues and patterns of thought (38).

Working memory accesses LTM for retrieval of prior knowledge in order to undertake tasks involving pattern recognition (39). Recent cognitive neuroscience models suggest that when new clinical information fits an existing schema, it can be integrated more efficiently, reducing cognitive effort. In contrast, unfamiliar or conflicting information demands greater cognitive effort, prompting clinicians either to develop new schemata or to search extensively for previously established schemata that are sufficiently similar and applicable to interpretation (40). Additionally, if information in long-term memory has been poorly encoded, remains fragmented, or lacks meaningful integration into relevant schemata, retrieval becomes less accurate (41, 42) and this may likely impair the clinician's ability to connect new information.

### Cognitive load theory

Cognitive load refers to the cognitive requirement for processing, integrating, and managing information as a conscious process (within WM) and is constrained by limitations in working memory capacity.

Cognitive load arises from the interplay of several components (43). Intrinsic cognitive load refers to the inherent complexity of a task, such as synthesising clinical data and processing it through multiple likely hypotheses at a single point in time (44). Extraneous cognitive load requires the individual to filter out irrelevant or distracting information, a process which itself imposes an additional cognitive burden. Within a clinical environment, this refers to the cognitive effort expended in filtering out irrelevant information imposed by distracting elements or aspects of patient history and data, that do not directly relate to established and relevant schemata (45). Germane cognitive load refers to the cognitive effort directed toward accessing, constructing and refining already established schema (46) when faced with new experiences or learning. While later conceptualisations (47) suggest that germane load may be better understood as a component of intrinsic cognitive load, rather than a distinct category, this article adopts the traditional tripartite model. Adopting a perspective including germane load as a separate category provides greater conceptual clarity for examining how clinicians engage in meaningful schema construction and refinement during diagnostic reasoning.

In clinical practice, excessive intrinsic cognitive load can hinder the ability to maintain, evaluate, and refine multiple diagnostic hypotheses. This is due to the confines of working memory being limited to retaining approximately 4–5 discrete items (5) or concurrent processing streams representing differential diagnoses, at any given time, while also being transient in nature. This is especially evident under time constraints or high-pressure conditions (48). However, even in less stressful environments, such as a family physician consultation, this limit on processing streams remains, underscoring the universal nature of this cognitive constraint.

#### AI and intrinsic cognitive load

AI systems, not being subject to the same cognitive load limitations, possess an unparalleled capacity for managing and processing vast amounts of information concurrently (49) with AI systems able to simultaneously process thousands of concurrent streams. This capability enables AI to effectively mitigate the limitations of human working memory, allowing it to represent a far broader spectrum of differential diagnoses (50), enhancing the diagnostic process by maintaining and evaluating a significantly larger array of hypotheses simultaneously.

Interestingly, some forms of AI also have intrinsic load and temporal decay related limitations with large language model-based AI systems, instead of experiencing gradual temporal decay as in human cognition, have a hard cut-off when a token (or context) window is exceeded (51). The token window is a rolling buffer that defines how much past conversation or text the model can consider, with a token being a full word, part of a word, or even a single character. Once an AI model exceeds its token window then earlier parts of a conversation or task are discarded—unless explicitly stored and recalled (52). Future architectures may integrate adaptive memory mechanisms to allow for more efficient retention, likely with the development of hybrid decision models, potentially utilising LLMs with other non-generative models (53). However, current LLM-driven diagnostic systems inherently possess these limitations, which should be explicitly acknowledged to maintain awareness and ensure continuity.

#### The human attentional control system

The human attentional system is highly adept at maintaining focus on tasks and efficiently filtering out irrelevant sensory information (54). Typically, only potentially relevant features, or that of suitable intensity, enter working memory, where they may contribute to extraneous cognitive load (37, 55). Most sensory information fails to meet the threshold of intensity required to bypass this attentional filter and is lost. In contrast, AI may approach this differently, not by filtering sensory information, but by processing a far broader range of inputs to identify the best fit for a central hypothesis or a series of differential diagnoses. This could be advantageous, as AI would have the potential to detect sensory and other information that might otherwise be lost during human attentional filtering. This is perhaps opposed to the human experience within a clinical setting, where a physician's attention might focus on the most salient or expected symptoms, potentially missing subtle sensory cues that could inform a different diagnosis.

Attentional control in a clinical context involves a delicate interplay between top-down and bottom-up processes (56), both of which physicians must balance to provide accurate and timely care. Top-down control is guided by the clinician's goals, expertise, and expectations, enabling focused attention on what is presumed to be most relevant. By contrast, bottom-up attention is driven by salient or unexpected stimuli, alerting a physician to potentially critical signals that might otherwise go unnoticed (57). Skilled practitioners navigate these dual processes by constantly switching between hypothesis-driven reasoning and reflexive awareness of clinical cues, thus optimising patient assessment (58). In comparison, AI systems use computational algorithms that, rather than selectively filtering data through human attentional constraints, can simultaneously process vast amounts of information (59) as input (bottom-up) while running internal processing streams (top-down) and while establishing their relevance through probabilistic analysis.

#### AI and extraneous cognitive load

Extraneous cognitive load arises from processing irrelevant or poorly presented information, imposing unnecessary mental effort on clinicians. AI appears at first sight to have some advantages in respect to processing extraneous cognitive load. AI systems have been demonstrated to be particularly adept in prioritising relevant clinical information and extracting meaningful patterns from large datasets, by algorithmically filtering out noise and highlighting only diagnostically salient features (59, 60).

These capabilities should translate into an ability to discern relevant from irrelevant information within a clinical scenario and thus would enhance diagnostic accuracy and alleviate cognitive burdens on clinicians (61). However, recent studies indicate that when exposed to real-world, uncurated data, LLM systems can perform relatively poorly. This performance drop can paradoxically add cognitive burden by generating misleading suggestions that clinicians must verify or override (53). In this respect, hybrid decision-support architectures, potentially combining LLM systems with Bayesian models (62) and with the clear necessity for maintaining human oversight, may offer a solution to these challenges.

It is noteworthy to mention that the capability of AI systems in respect to a fast-paced, multimodal and multifaceted clinical environment is unclear, with AI capabilities in filtering extraneous content under these conditions, not to our knowledge having been investigated at this juncture.

#### AI and germane cognitive load

Germane cognitive load refers to the cognitive effort directed toward accessing, interpreting current inputs, and refining existing schemata or constructing new schemata, when faced with new experiences or learning (43). With respect to germane cognitive load, AI systems trained on clinical data can build sophisticated representations of disease patterns, pathophysiological mechanisms, causality and diagnostic relationships. These models can be used to support clinicians in recognising and interpreting complex clinical presentations, effectively acting as external schema repositories (63). This would offer considerable superiority over limited access provided to schema through human working memory with its inherent bottleneck restricting processing to 4 ± 1 concurrent streams (5). AI systems would potentially be able to access thousands of schema-like representations (SLRs) simultaneously and rewrite information stores accordingly based on updated experiences, in real-time. These newly adapted SLRs would be compared with others, on a rather rapid timescale, thus refining accuracy and leading to new learning and improved diagnostic accuracy. These models offload much of the mental effort clinicians would otherwise expend in germane load during diagnostic reasoning and offering a clear advantage over human capabilities.

However, unlike in human cognition, where schema refinement is a bidirectional process involving constant modification through new experiences, current clinical AI tools are usually trained offline and deployment with a fixed dataset and thus apply these pre-existing models to interpret data without modifying them in real-time (64). Advancements in adaptive learning algorithms and the development of sophisticated AI models, likely using Bayesian networks, may enable AI systems to dynamically update and refine aspects of their internal schemas, mirroring the two-way schema modification seen in human cognition (65) and supporting more flexible and context-aware clinical reasoning.

### Cognitive models of reasoning

Diagnostic reasoning can also be understood through the lens of cognitive psychology, which examines how individuals process information and make decisions. According to Norman and Eva (66), diagnostic reasoning involves two primary cognitive processes: pattern recognition and analytical reasoning. Pattern recognition allows clinicians to identify familiar clinical presentations based on prior experiences, while analytical reasoning involves a more deliberate and systematic approach to problem-solving, often requiring the application of medical knowledge to solve unfamiliar problems (67).

#### The dual process theory

Various models of clinical reasoning have been proposed to account for the interplay between intuitive and analytical processes. The dual-process theory, a widely accepted model in cognitive psychology, provides a framework for understanding how the brain navigates these domains by categorising thinking into two systems. *System 1*, or intuitive reasoning, operates automatically and rapidly, drawing on pattern recognition from accumulated experience (68). It is predominantly an unconscious automated process enabling clinicians to recognise familiar clinical presentations and act with speed and efficiency (69). System 1 thinking dominates daily decision making where one is constantly engaged in making unconscious decisions. Heuristics which are mental shortcuts that allow decisions to be made without engaging in explicit, stepwise analytical reasoning, play a crucial role in this intuitive process. This type of reasoning is especially advantageous in high-pressure situations requiring quick decisions but is susceptible to cognitive biases when misapplied (68, 70).

In contrast, System 2 is slower, more deliberate and explicit, engaging the prefrontal cortex for the process of analytical reasoning (71). It involves critical evaluation, hypothesis generation, and ideally the integration of an explicit evidence-base. System 2 is essential when faced with novel or complex patient cases, requiring methodical problem-solving and consideration of differential diagnoses (69). Diagnostic reasoning frequently involves an interplay between these systems, where initial intuitive judgments from System 1, transition to System 2 for validation or refinement (72). This dynamic interaction highlights the importance of metacognitive awareness in knowing when to shift from intuition to analytical reasoning, based on the degree of perceived certainty in a hypothesis (69).

Experts leverage these dual processes more effectively than novices, achieving a dynamic equilibrium between implicit and explicit reasoning. Clinical experience allows the brain to consolidate vast amounts of clinical knowledge into patterns easily recognised by System 1, while still maintaining the capacity to engage System 2 for rigorous analysis when needed. This integration enables experts to move fluidly between intuitive and analytical reasoning, enhancing diagnostic accuracy and decision-making. In contrast, novices tend to rely predominantly on System 2, engaging in explicit processing that requires more cognitive effort and metacognitive monitoring. With the latter critical for novices for development of a better understanding of their reasoning processes and the accuracy of their conclusions (73).

AI systems operate under a fundamentally different paradigm compared to human cognition, particularly in the context of clinical decision-making. Human memory, as conceptualised by Tulving, is divided into episodic and semantic memory (74). Clinical, context-rich experiences, initially encoded as episodic memories, are thought to undergo semanticisation and are stripped of contextual details and consolidated into long-term memory, resulting in intuitive knowledge that facilitates rapid decision-making but lacks access to detail and stepwise reasoning (75).

While human cognition depends heavily on intuition for rapid processing and decision-making, AI, by contrast, leverages vast computational speed, capacity, and adaptive learning capabilities, negating the need for an analogous rapid and intuitive mechanism. AI systems can explicitly process and integrate information across multiple contexts without losing access to detail, allowing them to overcome the limitations introduced by semanticisation while maintaining rapid retrieval.

In principle, AI systems should be able to operate with transparent algorithms that can be audited and refined (76). A common criticism of many current deep learning models is that they remain largely as “black boxes” and offer limited insight into their internal decision pathways (20, 22), Current research into explainable AI (xAI) is attempting to bring more transparency to these models (77). Greater transparency in AI outputs enables systematic examination of the decision-making process, helping to reveal how specific inputs produce particular outputs and facilitating the identification and correction of biases, a level of transparency that will likely remain hidden in human system 1, or combined system 1–2 based reasoning (59).

Interestingly, recent AI research has increasingly incorporated aspects of dual-process theory into their system design (78, 79). Researchers developing dual-process AI models aim to combine the complementary strengths of intuitive, rapid pattern-recognition (System 1) and deliberate, analytical reasoning (System 2) into cohesive systems. While dual-process AI architectures have gained attention for aligning with human cognitive strategies, one might question their necessity computationally, given AI's capacity to perform extensive systematic analysis rapidly without need for heuristics, unlike in human cognition (80).

Nevertheless, such approaches still have relevance, particularly in clinical contexts, where dual-process AI through more accurately accommodating true human cognitive strategies, enables clinicians to interact seamlessly with both rapid intuitive outputs (System 1 friendly) and detailed analytical reasoning (System 2 based). Thus, clinicians can initially rely on AI-generated intuitive summaries, through abstraction (81), to quickly grasp complex patient data and then engage interactively with more detailed analytical explanations when deeper scrutiny or validation is needed. This structured yet flexible interaction effectively mirrors clinicians' natural cognitive processes (72), making AI outputs more usable and cognitively accessible.

Further discussion on the role of dual-process systems in AI has focused on the interplay of systems 1 and 2 in the generation of a metacognitive process (82). This approach attempts to mirror human metacognition (83) and involves incorporating a metacognitive layer that monitors and regulates the interaction between intuitive and analytical processes. However, since AI systems do not require heuristic shortcuts for rapid decision-making, the imposition of dual-process metacognitive structures in this manner, may introduce unnecessary complexity without offering any functional advantage beyond a potential decrease in computational load.

#### The role of heuristics in diagnostic reasoning

Pattern recognition is a widely used form of diagnostic decision-making, particularly among experienced clinicians. It involves the rapid identification of familiar clinical presentations based on prior knowledge and experience (84), allowing for swift decisions without the need for extensive analytical reasoning. Aligning with the intuitive and automatic processes characteristic of System 1 thinking, this process is predominantly rooted in use of heuristics (72, 73). Heuristics are cognitive shortcuts or rules of thumb that often operate unconsciously. For example, using a heuristic a clinician might recognise a symptom set such as chest pain radiating to the arm as indicative of the onset of myocardial infarction, even if they cannot consciously articulate every component of the reasoning process (69).

Heuristics allow clinicians to make quick decisions based on limited information, and enable judgments under uncertainty (85) by leveraging pattern recognition, prior experiences, and semanticised schemata. Unlike conscious analytical reasoning (system 2), these schemata operate automatically, enabling rapid decision-making, even when components of the underlying diagnostic algorithm are incomplete, and often without the clinician's conscious awareness of how the schema was formed or why it applies. Most importantly, the specific components of heuristic-based decision-making are challenging to delineate within distinct decision arcs, as much of the process has become semanticised, making it less accessible to conscious introspection. In this respect, it will be unknown whether these shortcuts are incomplete or flawed. Thus, while heuristics enhance efficiency, they also introduce vulnerabilities (85), and can lead to errors when these non-declarative forms of schema are incomplete, inaccurate, key data is missing or are shaped by cognitive biases (68).

It is important to note the role of System 2 thinking as an analytical process that, although slow and effortful, can override or refine intuitive judgments originating from System 1. In clinical settings, for example, anchoring-and-adjustment (86, 87) typically begins with a System 1 anchor, an initial diagnosis formed intuitively. System 2 then critically evaluates this anchor and adjusts it based on new evidence or additional clinical data. Engaging in such deliberate, evidence-based reasoning (68) is characteristic of System 2 cognition, being particularly critical for reevaluating initial impressions, integrating broader clinical evidence, and reducing cognitive biases that might otherwise compromise diagnostic accuracy (83).

#### The role of AI in addressing heuristic limitations

Artificial intelligence (AI) systems can mitigate the limitations of heuristic-based reasoning by offering structured and systematic approaches to diagnostic reasoning. Unlike human cognition, relying on schemata that may be incomplete, biased or partially inaccessible due to cognitive limitations, AI systems can consistently apply probabilistic models and causal reasoning (63), even when key elements of a schema are missing or unclear.

Despite the opacity of many deep learning systems due to hidden layers and large parameter counts, emerging explainable AI (xAI) models may offer greater transparency in diagnostic applications. Given the potential capacity to provide transparent chain-of-thought reasoning, explainable AI can serve as a powerful tool to deconstruct and elucidate the components of heuristic structures (21, 76). Explainable AI through detailing components, similarly to disclosing a more complete algorithm, it can potentially highlight and correcting inaccuracies within heuristic frameworks, thus mitigating potential cognitive biases and enhancing decision accuracy. A practical example would involve an xAI based model processing patient data and, through the application of probabilistic models, causal reasoning, and close adherence to latest clinical guidelines, identify alternative diagnostic possibilities that challenge or extend beyond the clinician's initial heuristic judgment thus prompting further clinical investigation (63).

While such scalability permits AI to detect patterns that might be overlooked by a cognitively overburdened physician, it is widely proposed that it may also miss contextual nuances that human intuition captures (22). Despite these concerns, it is important to recognise the potential of advanced AI models, when trained extensively across diverse, real-world contexts, with their ability to store enormous amounts of data, their sheer processing capacity and potential to systematically evaluate vast numbers of data streams, they could theoretically process explicit data within contextual frameworks in a manner similar to human intuitive perception. Although this capability has not yet been fully demonstrated, it remains a plausible direction for future development.

#### Human cognitive biases

Cognitive biases often arise when System 1 dominates decision-making because of its reliance on heuristics. While heuristics can be useful, they can also lead to predictable errors when information is incomplete or ambiguous, when emotions, past experiences, or social influences interfere, or when there is pressure to make quick decisions (88).

O'Sullivan and Schofield (70) emphasise the prevalence of several biases that commonly affect diagnostic reasoning. Two of the most common are, *anchoring bias* where clinicians fixate on an early impression and fail to adjust their judgment as new information becomes available (86) and *availability bias* occurs when recent or easily recalled cases disproportionately influence judgment (89).

*Confirmation bias* occurs when clinicians favour information that supports their initial diagnosis while dismissing contradictory evidence (90). *Representativeness bias* (86) which reflects a tendency to diagnose based on a patient's resemblance to a prototypical case with typical or “classic” features of a condition, while failing to account for its actual prevalence in the population (91). Other biases include *overconfidence bias*, where clinicians overestimate their diagnostic accuracy or capability (92), and *premature closure*, which involves halting the diagnostic process once a plausible explanation is found, despite the diagnostic method being insufficient (73).

In these situations, system 2 reasoning is particularly critical for reevaluating initial impressions, integrating broader clinical evidence, and consciously addressing potential cognitive biases that might otherwise compromise diagnostic accuracy. However, in high pressure clinical situations the opportunity for engaging in such deliberate, evidence-based reasoning may not be available. While biases are often associated with heuristics and System 1 operations, System 2 thinking can also accommodate biases, especially when clinicians rely on incomplete or incorrect schemata, which can distort the interpretation of clinical information (93).

#### The role of AI in mitigating human bias and managing Its Own

AI systems have the potential to assist in mitigating certain cognitive biases inherent in human clinical reasoning (94) systematically addressing these limitations through providing probabilistic analyses, free from human error. By systematically analysing the entirety of available data, AI avoids cognitive shortcuts leading to anchoring bias in humans, ensuring that all relevant possibilities are evaluated even when critical data points are absent (95). This approach highlights the potential for AI to also act as an essential adjunct in clinical decision-making, particularly in complex cases or where system 2 reasoning may falter due to data insufficiency, cognitive overload or the presence of inaccurate schemata. While AI systems can assist in minimising certain cognitive biases in the immediate term, their effectiveness would be further enhanced through human interaction with explainable AI interfaces. Such interaction not only promotes cognitive debiasing by increasing transparency, potentially mitigating an individual's habitual reliance on biased reasoning patterns, but also supports the correction of faulty schemata and heuristics through the presentation of interpretable and contextually accurate information.

It is important to note that biases can also be present in AI diagnostic systems. *Data bias* where AI algorithms trained on historical clinical data, may reflect existing human biases (96), where if the training data are unrepresentative or skewed toward certain populations, AI systems may produce biased outcomes that exacerbate health disparities (97). *Algorithmic bias* occurs with the design and implementation of AI algorithms where the selection of features used for training or the decision thresholds are set by developers based on flawed assumptions (98). Here such algorithms may prioritise certain clinical indicators over others.

An interesting study by Vicente and Matute (99) describes a *transfer of bias* phenomenon where individuals who are exposed to biased AI outputs may carry those biases into their independent decision-making processes. This transfer effect can perpetuate errors and reinforce existing biases in clinical settings, even when the AI system is no longer involved.

These forms of bias within AI system require careful management and undoubted human oversight at both data stewardship and the output stage. However, with improved data and algorithms programmed to enforce appropriate representation, these can be largely mitigated (100).

#### Bayesian reasoning

Bayesian reasoning in human cognition is fundamentally an iterative process of updating cognitive structures where the mind begins with a set of prior beliefs or mental models (*priors*), shaped by past experiences, innate predispositions, and learned principles. When new information or evidence is encountered, the mind evaluates it against *priors*, weighing how much it aligns with or contradicts existing assumptions (101). This comparison leads to an updated mental model, the *posterior model*, which integrates both the prior knowledge and the new evidence. Griffiths and Tenenbaum (102) highlight that the ability to combine accurate background knowledge with systematic statistical reasoning is critical in many aspects of higher-level cognition, suggesting that prior beliefs significantly influence how new information is processed.

This iterative nature of Bayesian reasoning allows individuals to refine their mental models progressively, ensuring that they do not overreact to novel evidence while also avoiding the pitfalls of rigid adherence to outdated assumptions (103). Bayesian reasoning accommodates uncertainty by allowing beliefs to be held with varying degrees of confidence, which is influenced by the strength of prior knowledge and the quality of incoming evidence (104). This dynamic equilibrium achieved through this continuous revision of mental models enables individuals to adapt to complex and ever-changing settings (105) and is particularly pertinent to the clinical environment.

Human cognitive architecture does not naturally lend itself to perfect Bayesian updates due to cognitive limitations. Bayesian inference (a formal, mathematical framework based on Bayes' theorem in its' ideal form, assuming extensive computation and perfect rationality) demands tracking of multiple conditional probabilities in real-time. However, human working memory has clear capacity limitations (5) which makes it difficult to calculate or represent all relevant probabilities simultaneously. As a result, individuals approximate or collapse complex information into more manageable chunks, often losing fine detail in the process.

Furthermore, to cope with such cognitive limitations, human cognition often relies on heuristics, with their complications constrained by various biases (86), with representativeness bias particularly relevant, being driven by resemblance to a mental prototype, rather than considering the unknown or accurately weighing new evidence. As a result, Simon (106) defines a *bounded rationality* which describes how people aim to make good-enough rather than fully optimal decisions. This phenomenon has been described extensively within clinical diagnostic reasoning processes (107).

Conversely, AI systems particularly those using Bayesian networks, following explicit rules for updating probabilities, should perform Bayesian inference more precisely than in human cognition due to their ability to manage large-scale computations without cognitive constraints (80). Their immense computational capacity should enable AI models, to manage vast numbers of variables and calculate posterior probabilities based on their extensive datasets. Algorithmic consistency also ensures that given the same inputs and priors, AI will generally repeat the same output after taking the observed data into account. Conversely, human cognition may respond inconsistently under different contexts or levels of cognitive load, or from emotional or motivational influences that can skew human reasoning.

However, it is important to note that current large language models (LLMs) do not truly perform Bayesian updating. Instead of continually adjusting their beliefs as new information comes in, they rely primarily on pre-trained parameter relationships and struggle with formal probabilistic reasoning. Recent studies (15) have reported that despite excelling in many clinical reasoning tasks, LLMs like the OpenAI o1 platform perform poorly compared to human clinicians on tasks requiring accurate probabilistic inference. These models often exhibit flawed understanding of conditional probabilities and do not reliably adjust likelihood estimates with new evidence, highlighting an important limitation in their capacity for reasoning under uncertainty.

#### Reasoning under uncertainty

Human reasoning under uncertainty is inherently adaptive, shaped by a combination of prior knowledge, heuristics, and probabilistic inference (chiefly Bayesian). However, this process is shaped by cognitive biases as described and individual differences in metacognitive sensitivity, an individual's ability to accurately assess their own cognitive performance, and metacognitive control, involving strategic mechanisms by which individuals regulate their cognitive processes to optimise learning and decision-making (108, 109). When these metacognitive functions operate well they can enhance clinical reasoning, or when operating poorly can contribute to diagnostic errors.

With human reasoning susceptible to inconsistencies, particularly when faced with novel or complex scenarios, Bayesian capabilities represented in human cognition, are subject to the confines of limited working memory capacity, thus limiting the range of hypotheses or analytical steps that can be explored simultaneously (110).

Conversely, artificial intelligence systems, offer a more structured and systematic approach to reasoning under uncertainty, with the capacity to make judgments even in the presence of missing data (111). AI leverages probabilistic models, such as Bayesian networks as well as others, to infer the likelihood of outcomes based on known variables while accounting for the absence of certain inputs (112). For example, when patient data is incomplete, these models estimate the probabilities of various diagnoses using the relationships between observed data points (113) while providing explicit probability scores (80).

Thus, AI systems are likely better able to engage in analysing diverse datasets and applying probabilistic algorithms, with vastly increased throughput over human cognitive limitations, thus offering unique advantages in handling complex or incomplete information and underscoring their potential to complement and enhance human diagnostic processes.

#### Contextual factors in diagnostic reasoning

Contextual factors, such as time pressure, physician fatigue (114), with prolonged duties impacting vigilance (73), a disorganised or resource-limited work environment, decision fatigue (115), and personal stressors or emotional difficulties unrelated to patient care (116) can significantly impact diagnostic reasoning performance. Stress and anxiety have been shown to compromise working memory by reducing attentional control, leading to diagnostic inefficiencies (117, 118). Similarly, cognitive fatigue, which arises from sustained mental demand during prolonged shifts or high-pressure situations, can further reduce working memory capacity, slowing processing speed and impairing accuracy (119).

AI systems have the potential to mitigate these challenges by providing consistent support during diagnostic tasks. Unlike human clinicians, AI systems are likely unaffected by stress or fatigue (120), enabling them to maintain accuracy and speed even in high-pressure or prolonged-use scenarios (22). Critically, AI may be able to assist in managing the effects of such interference by acting as a second opinion, helping to validate or challenge diagnostic decisions impacted by stress, emotional impacts or cognitive fatigue. In this way, AI complements human diagnostic reasoning by providing a stable and objective framework, ensuring more consistent performance across varying contexts.

Interestingly, AI systems although not subject to biological fatigue as such, can experience performance degradation over time due to memory saturation, where the system's context memory becomes overloaded, reducing coherence and output quality (121). In such cases, a reset or memory clearing operation can restore optimal performance, serving a role loosely analogous to the restorative function of sleep.

### Models of diagnostic reasoning

**Figure 1:**
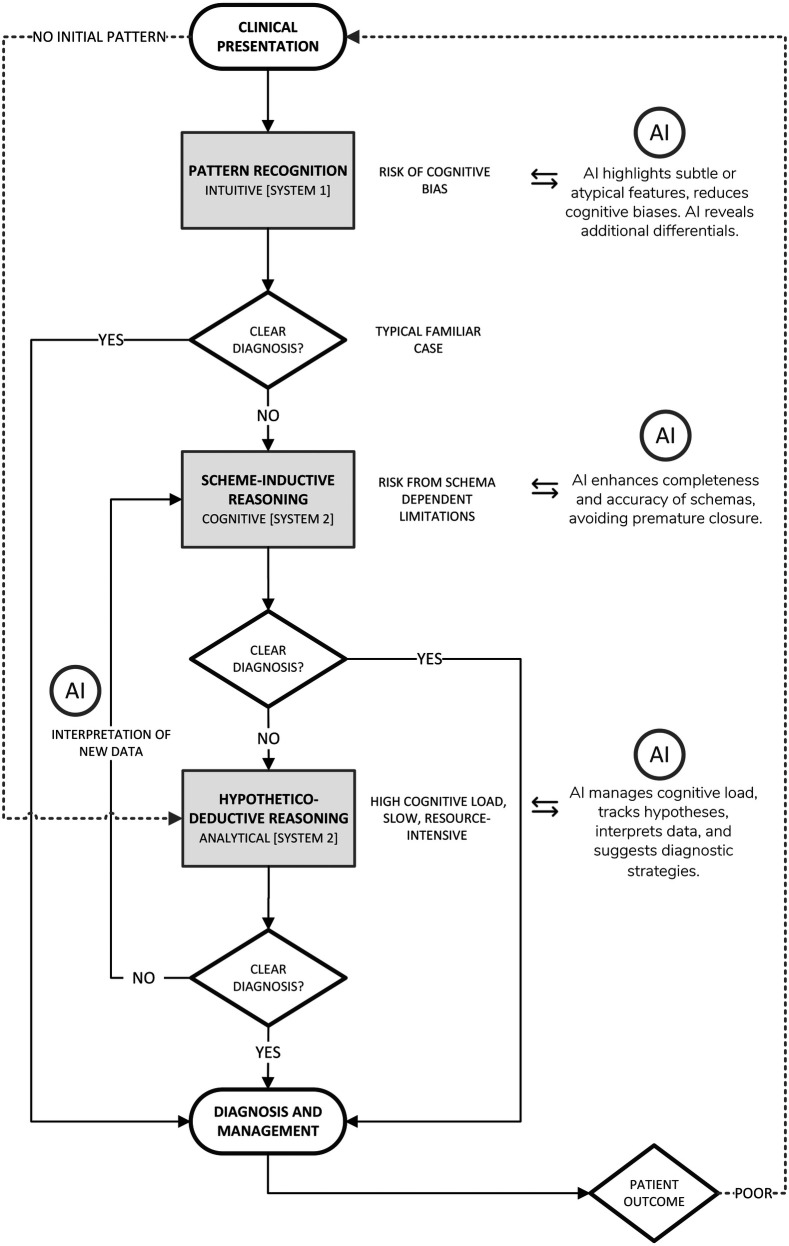
Diagrammatic representation of the diagnostic reasoning process with opportunities for AI-driven support.

Clinical diagnosis characteristically oscillates among three cognitive strategies; Pattern Recognition, Scheme-Inductive Reasoning, and Hypothetico-deductive Reasoning (Figure 1). Each strategy engages different levels of Bayesian reasoning, requires a distinct cognitive-load profile, and presents specific opportunities for AI augmentation.

#### Pattern recognition

The most commonly utilised diagnostic reasoning model for experienced practitioners is pattern recognition. Pattern recognition is a rapid, intuitive (System 1) process. It is largely automatic, unconscious, and experience-driven**,** thus allowing the rapid identification of familiar clinical presentations based on prior experience (84) with immediate consideration of management strategies (likely also as an unconscious process). Triggered by just a few salient cues, usually from symptoms and patient history, which may closely match a previously encountered presentation, these are represented in a clinician's pre-existing, highly compressed *illness script* held in semantic memory. Pattern recognition proceeds almost instantaneously, providing the clinician with an automatic, almost entirely intuitive idea of the likely diagnosis (68). Pattern recognition is predominantly governed by heuristics and thus prone to cognitive biases. Illness scripts function as implicit Bayesian priors where the clinician subconsciously assesses that the posterior probability of the familiar diagnosis is high, without explicitly calculating any real probability ratios.

The major liabilities of pattern recognition, given its reliance on heuristics, are anchoring, availability bias, and premature closure, particularly when atypical or low-prevalence conditions present with some similarity to common patterns. This leads to contextual or less salient data being ignored if they do not fit the prototype (4). Moreover, pattern recognition often encourages immediate commitment to the first illness script that matches salient cues, accepting the first plausible match (73) which as a result may overlook other possibilities. Once each match is accepted, being an unconscious process, this does not provide for any significant cognitive dissonance (discomfort thus prompting a search for competing explanations) (68), nor are metacognitive processes employed, so the cognitive system closes early and any decision remains unquestioned. Empirical studies of diagnostic error consistently list premature closure as the most frequent cognitive cause, which is related predominantly to inaccurate pattern recognition (122).

Because pattern recognition utilises chunked information, and heuristics, intrinsic working-memory (WM) load is negligible (123). As a result of this low cognitive burden, fatigue exerts relatively little influence and this form of reasoning is most often a default under conditions of stress and fatigue (73).

##### The impact of AI on pattern recognition

AI-enabled clinical decision support systems can act to provide independent verification, analysing the same presenting features that triggered pattern recognition but comparing them rapidly with vast amounts of clinical data. Thus, the CDSS is able to either confirm that the clinician's initial match is statistically well supported; or indicates that the presentation more closely resembles an alternative, that may have been overlooked (22). Using explainable-AI models, with visualisations such as Grad-CAM heat-maps (124), outputs incorporating explicit Bayesian probability scores, or natural-language explanations generated by large-language models (125) can clearly explain why the algorithm supported or rejected particular diagnoses.

To mitigate cognitive vulnerabilities inherent in pattern recognition, some Electronic Health Record dashboards include diagnostic time-outs. This form of feedback invites the clinician to pause and reflect, promoting metacognitive awareness and potentially countering the tendency towards premature closure, guiding clinicians to revisit assumptions, explore alternative pathways also consistent with the presentation, and ultimately reduce the risk of diagnostic failure (126, 127).

Ideally, an AI-driven CDSSs would build on these principles by detecting behavioural markers of premature closure, such as rapid narrowing of the differential diagnoses, failure to integrate new or discordant findings which disagree with the working diagnosis, or ceasing further investigations (72, 122). Subsequently, within the same dashboard interface, such detection could automatically trigger a prompt to initiate a diagnostic pause and re-evaluate alternative explanations before progressing further. By acting as real-time metacognitive support, while providing advice on missing strategies, these modules would help maintain diagnostic vigilance and potentially help clinicians recognise and correct their diagnostic habits.

Other diagnostic reasoning models such as scheme-inductive and hypothetico-deductive reasoning form the backbone of most clinical decision-making, outside pattern recognition, offering structured approaches to synthesising complex patient data (84). While these models provide a more systematic framework, they are also not without limitations. Both approaches rely heavily on human cognitive resources, with their limitations described earlier, presenting opportunities for artificial intelligence systems to intervene. The following discussion examines these models and how AI can augment their application in clinical practice.

#### Scheme-inductive reasoning

In cases where pattern recognition yields uncertainty (see Figure 1), clinicians typically engage in the conscious, system 2 based process of scheme-inductive reasoning. Scheme-inductive reasoning in clinical diagnosis is a structured deliberate and conscious cognitive process facilitating forward reasoning from symptoms to diagnoses, where clinicians recognise more detailed patterns in patient presentations and apply pre-existing knowledge frameworks (schemata) to generate and refine differential diagnoses (128).

Cognitive architecture underpinning schema-based reasoning involves a schema, representing a structured framework of organised, encapsulated knowledge (or detailed illness script), which when activated and retrieved from long-term memory, guides diagnostic reasoning by organising causal relationships within clinical scenarios and aiding clinicians in quickly structuring and interpreting clinical information (129).

Unlike heuristic-based methods which rely on intuitive pattern recognition and may introduce cognitive biases, scheme-inductive reasoning largely structures knowledge explicitly providing a cohesive analytical framework where the diagnostician compares patient data with a stored schema to confirm a diagnosis and implement management (130). For example, when encountering a patient with hypertension and high cholesterol presenting with sudden, crushing chest pain radiating to his left arm, this would activate the *Acute Coronary Syndrome* schema in the clinician's mind and guides them to prioritise this diagnosis over others. This pre-existing mental framework contains detailed information on typical presentations, pathophysiology and inherent causality, risk factors, and management (130).

Scheme-inductive reasoning aligns with the natural cognitive processes of the human brain, enabling clinicians to organise complex information into manageable units or “chunks” (123) and to focus on the most relevant causal links within a specific diagnostic context (131). By leveraging pre-existing schemata, clinicians can efficiently navigate complex cases (69, 132), reduce cognitive load (133), and improve diagnostic accuracy (84, 128). Here, a structured diagnostic schema operates like a Bayesian decision tree where prior probabilities are embedded in the branch architecture, the clinician mentally traverses along the tree, while reflecting on the relative likelihood of downstream nodes or branches and updating the probability of each branch having relevance as new evidence arrives (128).

Importantly, given that scheme-inductive reasoning, guides an inductive process to formulate a list of possible conditions based on the activated schema, the previously defined schema is constantly refined with new insights, enhancing diagnostic accuracy for future cases. A further advantage of scheme-inductive reasoning lies in its ability to align with the natural way the human brain organises information. By leveraging schemata and narrative structures, clinicians can manage complex data, focusing on the most relevant causal links, thus structuring a case in a way that enhances communication, by providing the utility of a shared narrative within a clinical team (131).

##### Cognitive limitations related to scheme inductive reasoning

Although, scheme-inductive reasoning offers a structured and efficient framework for clinical diagnosis, it is inherently susceptible to several limitations of human cognition. While highly effective, scheme-inductive reasoning is cognitively demanding, particularly for novice clinicians, who often lack the extensive repository illness scripts that experienced clinicians draw upon (134). The limited capacity of working memory (5) restricts the number of diagnostic hypotheses a clinician can actively consider simultaneously. Moreover, where it relies heavily on the completeness and accuracy of existing schemata, those stored in long-term memory, may be partially incorrect, incomplete, outdated, inefficient, influenced by cognitive biases or corrupted by interference, thus compromising diagnostic accuracy (129).

Inefficient organisation of schemata with extraneous largely unrelated branches within the structure, can increase cognitive load. A diagnostic schema generates a degree of intrinsic cognitive load at each branching decision point, while the need to maintain viable alternative diagnoses in working memory imposes an additional cognitive demand (133). In addition, as reasoning chains grow longer, this amplifies the risk of temporal decay and cognitive fatigue. Finally, although more structured and explicit than pattern recognition, if clinicians overly rely on familiar patterns this can still lead to premature closure, potentially disregarding critical clinical data that do not align closely with existing schemas (4, 130).

#### Hypothetico-Deductive reasoning

The Hypothetico-deductive reasoning (HDR) model (Figure 1) is a foundational approach to clinical problem-solving and is particularly useful in allowing for a structured exploration of diagnostic possibilities. HDR is similar to building a case from the ground up and systematically guides clinicians through iterative cycles of “generate hypothesis → order/interpret test → refine hypothesis → repeat”, thus gathering evidence, considering diagnostic possibilities, and then systematically testing each one until arriving at the most likely explanation. HDR has been widely recognised as a core element of clinical reasoning and has been extensively studied in medical education and practice (134, 135).

Hypothetico-deductive reasoning relies principally on Bayesian principles, whereby clinicians generate initial diagnostic hypotheses (priors) based on available patient data. These hypotheses are then tested and iteratively refined as new clinical evidence becomes available, with probabilities updated to reflect changing levels of diagnostic certainty (136). Although this process can be construed within Bayesian principles, clinicians typically apply it either heuristically or from preestablished schema rather than actually calculating probability values. This cyclical process of adding evidence, further evaluation and hypothesis adjustment continues until the differential narrows and the most probable diagnosis is identified. Based on this outcome, the clinician proceeds to formulate a treatment plan, carefully weighing risks and benefits within the context of the patient's condition and their level of diagnostic confidence. Hypothetico-deductive reasoning under uncertainty is also characterised by clinicians initially collecting comprehensive patient data, including symptoms, history, and physical exam findings. In the absence of clear hypotheses, this may begin with broad investigations to gather more data.

##### Interaction between scheme-inductive and hypothetico-deductive reasoning

Scheme-Inductive reasoning and Hypothetico-deductive reasoning typically interact dynamically (Figure 1). HDR provides the framework for systematically testing hypotheses, with their underlying content usually guided by intuitive processes such as pattern recognition or structured schema activation (within scheme-inductive reasoning), drawing upon the clinician's knowledge and mental models of diseases, or illness scripts (134).

Initially, clinicians activate diagnostic schemas to generate one or more structured hypotheses based on key clinical patterns. Subsequently, clinicians employ HDR to systematically test these hypotheses through detailed data collection and analysis. Once test results emerge, schema-based reasoning is reactivated to interpret these findings, thus refining hypotheses further or adjusting the schema to accommodate new insights (128).

Despite scheme-inductive reasoning having been shown to outperform traditional hypothetico-deductive reasoning in certain scenarios, particularly in terms of efficiency and diagnostic precision (4, 84, 128) this assertion may overlook that they typically function collaboratively in practice. Indeed, it may be challenging to isolate the benefits of scheme-inductive reasoning without considering its complementary role alongside HDR.

#### Cognitive Limitations in Hypothetico-deductive Reasoning

A primary limitation of Hypothetico-deductive reasoning lies again in the constraints of human cognitive capacity. Integrating large volumes of complex data produces substantial intrinsic cognitive load, while accommodating multiple competing hypotheses, places a significant capacity burden on working memory (67).

Compounding this is the phenomenon of temporal decay. This is particularly problematic in iterative diagnostic processes, where hypotheses must be continuously revisited and refined (135) with sequential data gathering over time. However, each new finding or test result competes for limited working-memory resources and introduces interference, effectively “overwriting” earlier hypotheses. This is compounded when combined with temporal decay, which causes earlier hypotheses to fade from working memory (137). This creates cognitive bottlenecks, as the limited capacity and time-sensitive nature of working memory prevents thorough exploration of all potential diagnostic options, increasing the risk of diagnostic oversight or premature closure. Additionally, unlike artificial intelligence, human cognition also has a rather limited processing speed, which increases the time required to evaluate and integrate disparate pieces of diagnostic information, especially in ambiguous or conflicting cases. As a result, clinicians may struggle to retain and process all relevant information simultaneously, particularly in complex cases.

Emotional and psychological factors further constrain HDR's effectiveness. Stress and time pressure, ubiquitous in clinical environments, can impair mental flexibility and exacerbate cognitive biases. Fatigue from long shifts or inadequate rest also diminishes memory, attention, and decision-making capacity, further hindering the ability to apply HDR systematically (93). In these conditions, clinicians may default to heuristics, potentially compromising diagnostic accuracy and introducing cognitive biases with anchoring bias being a common pitfall (93).

Errors in the iterative processes of HDR are another common limitation. Clinicians may exhibit confirmation bias during hypothesis testing, seeking evidence that supports their initial hypotheses while dismissing contradictory data. Decisions regarding diagnostic tests may also reflect over-testing (wasting resources) or under-testing (missing critical investigational data), both of which undermine the HDR process (138). These challenges highlight the fragility of HDR particularly when applied in the demanding and fast-paced clinical environment. Despite these weaknesses and that HDR is slow and resource-intensive, it remains indispensable for novel or complex cases, providing a more systematic coverage of the diagnostic space (135).

### Impact of AI on clinical reasoning

Recent developments in AI have introduced tools designed to support and augment human cognitive processes. This relationship is illustrated in Figure 1, which maps the diagnostic workflow and highlights how AI can complement various human reasoning strategies.

#### In refinement of the causal schema

In terms of addressing incomplete, outdated, or incorrect schema, advanced AI models can simulate causal frameworks and contextualise complex relationships within plausible narratives (63, 139), not only contributing to lowered cognitive load (140) but also providing a structure that complements the encoding form, represented within the newly activated clinician's schema, and thus facilitating efficient learning (38).

AI systems can enhance scheme-inductive reasoning where these models can dynamically link diagnostic data, clinical guidelines, and patient history, with a far greater capacity and speed as per human cognitive ability. Moreover, these systems, through an ability to present a correlated view of each case may be able to flag potential discrepancies within clinical reasoning, thus helping identify and correct faulty or outdated schemata stored in long-term memory, thereby offering the clinician an opportunity for diagnostic recalibration and further learning. Furthermore, within the AI derived output, by reorganising complex schemas, clustering related concepts and pruning low-utility branches, AI can reduce the cognitive burden associated with schema activation and traversal, thereby enhancing efficiency (141).

Moreover, when clinicians repeatedly retrieve and apply a schema with the inclusion of chain-of-thought outputs from AI, a form of explainable AI, schema (142) and associated retrieval cues (143) are further consolidated, strengthening memory access in the future.

Explainable AI systems may enable clinicians to trace each step and critically evaluate the AI reasoning process (144), but also to compare machine-generated outputs with their own schemata, identifying potential inconsistencies or errors on either side (145) thus providing further learning opportunities and improving metacognitive monitoring (109, 146).

Finally, the potential of advanced AI systems to dynamically update reasoning in response to new information (24, 65), similarly to updating cognitive schemata, ensures that diagnostic recommendations remain current and evidence-based, reducing the risk of outdated or incomplete reasoning.

#### The Use of AI in streamlining investigations

AI systems can undertake an iterative process that parallels hypothetico-deductive reasoning (95). With several emerging tools this process may become highly streamlined, via use of automated clinical investigations tightly integrated with advanced AI-driven diagnostic reasoning platforms (147), which can interpret data and then compile and analyse a large number of illness scripts for comparison simultaneously and in real-time. Technologically, cloud-based AI systems such as Google Vertex AI (148), Microsoft Azure Machine Learning (149), and Amazon SageMaker (150) have the notional capability to engage data ingestion, importing and processing data from various sources including patient monitoring systems. Some platforms such as NVIDIA Clara are already utilised in medical imaging and patient monitoring in ICU settings (151), and Siemens Healthineers' AI-Rad Companion operates in the medical domain for interpretation of medical imaging (152). These developments suggest that automated clinical investigations linked to sophisticated diagnostic reasoning tools, and incorporating a form of hypothetico-deductive reasoning, are on the cusp of wider implementation, promising much improved speed, efficiency, and accuracy in medical diagnostics.

#### AI in interpretation of results and schema updating

Bayesian engines can calculate posterior probabilities for each potential diagnosis and highlight other possible branches (128, 153), with continuous probabilistic feedback prompting clinicians to revisit neglected alternatives. Furthermore, they can provide dynamically updated schema-like structures with population-level data, highlighting key discriminators, and issuing probabilistic prompts when the reasoning trajectory risks closing prematurely (125).

AI-generated interactive dashboards displaying the patient data, active paths, plausible but dormant differentials, outstanding discriminators and their Bayesian probabilities are likely useful innovations. By externalising these data these may be utilised to decrease intrinsic cognitive load demands (154) and reduce extraneous load providing a filter to noise present in the diagnostic process. Moreover, the clinician can instead focus working memory resources on germane cognitive tasks (155).

Dashboards containing AI-generated saliency overlays on imaging and highlighting areas influential in each diagnosis, with attribution bars for laboratory variables, offer visual contributions to managing cognitive burden. Furthermore, this would enhance shared understanding, support clinical dialogue, improve transparency enabling automated outputs to be clearly interpreted by clinicians (124).

By presenting these outputs according to a stepwise, chain-of-thought approach, thus adhering to the narrative structure that reflects human cognitive processing (156, 157), AI systems enhance interpretability and support collaborative (human-to-AI) reasoning, reflecting principles underpinning the mutual theory of mind concept (158, 159). Furthermore, explainable AI systems may enable clinicians to trace each step and critically evaluate the AI reasoning process (144), but also to compare machine-generated outputs with their own schemata, identifying potential inconsistencies or errors on either side (145) thus providing further learning opportunities.

In conclusion, by integrating AI's capability to process vast amounts of data, engage in chain-of-thought reasoning, and dynamically adapt to new information with the principles of scheme-inductive reasoning, clinicians can achieve a more robust and accurate diagnostic process, leveraging the strengths of both human cognition and advanced technology.

Figure 1 presents an AI-augmented model of clinical diagnostic reasoning that integrates the three key human reasoning strategies; Pattern Recognition, Scheme-Inductive Reasoning, and Hypothetico-deductive Reasoning, highlighting targeted AI support at each stage. A patient's clinical presentation is first evaluated via rapid Pattern Recognition (System 1 based); if a clear diagnosis emerges, management follows. When no clear pattern is detected, the clinician employs the Scheme-Inductive Reasoning (System 2 and cognitive based), working through illness schemata and comparing discriminators; AI at this node enhances schema completeness (through xAI), highlights high-utility branches, and issues prompts to avoid premature closure.

Hypothetico-deductive Reasoning (System 2 and analytical) generates and iteratively tests competing hypotheses. AI here offloads the main cognitive burden by tracking hypotheses, calculating Bayesian probabilities, suggesting optimal investigations, and interpreting incoming data. At each failed “Clear Diagnosis?” decision point, dashed arrows guide the clinician onward, while successful diagnoses proceed to management. Reciprocal arrows reflect principles of the Mutual Theory of Mind (MToM), whereby the AI system models the clinician's knowledge and reasoning strategy, and the clinician interprets and responds to AI outputs.

This layered approach not only mirrors a clinicians' natural progression from intuition to analysis but also demonstrates how AI tools can reduce cognitive load, improve accuracy, and accelerate decision making throughout the diagnostic pathway.

### The mutual theory of mind

Incorporating a Mutual Theory of Mind (MToM) perspective (a bidirectional mental-modelling paradigm) into explainable-AI-driven clinical decision support systems could markedly enhance both human diagnostic reasoning and AI performance. MToM can be conceived as a continuous negotiation of mental models (160) between the clinician and the AI algorithm. While the clinician learns from AI, eventually AI will incrementally be able to develop an internal representation of the clinician's knowledge, goals and workflow while simultaneously exposing its own reasoning to clinical practice. This bidirectional modelling has been articulated conceptually (158) and has been attempted in empirical studies (159). As AI's capacity increases, it may extend to identification within the individual clinician, of cognitive biases (161), levels of metacognitive accuracy, attentional focus (detecting diurnal variations in performance), the robustness of visual (162) and diagnostic schemata, and even individual parameters of working-memory and cognitive load. This analytical approach to knowing the cognitive capacity of the clinician enables the CDSS to optimise its support specific to the clinician's individual cognitive profile. Simultaneously through the clinicians examination of transparent feedback loops on performance, this could potentially lead to improvement in the clinicians own metacognitive monitoring (146), where they may become more attuned to their own cognitive limitations.

### Ethical considerations

Adoption of AI into clinical decision support should not be isolated in terms of predictive power and accuracy. It is critical to ensure ethical frameworks accompany deployment and must satisfy core ethical requirements of being transparent, consensual, and aligned with individual patient values. Transparency requires that AI outputs are presented in an interpretable form that allows critical human oversight of diagnostic pathways and potential biases, rather than functioning as opaque “black boxes” (20, 21, 145). Consent is again critical, referring to informing patients of use of AI in diagnosis and treatment planning and communicating implications of the reuse of clinical data to train or improve AI models. For clinicians, this requires working within clear policies about when and how to tell patients that AI tools are informing diagnostic reasoning, choices patients have including any opt-out options (18, 29), and ensuring that decisions optimised at a population level do not override values and priorities of the individual (29). Finally, questions of data ownership and control over these training datasets are equally important with proposals such as the Global Patient co-Owned Cloud (GPOC) envisaging patient co-ownership of personal health records and shared governance over how such data are accessed and used for AI development (163).

### Further work

It is clear that at the current level of technological development, AI must be integrated as a collaborative tool that augments, rather than replaces, human reasoning (164). Although in initial studies, LLMs having demonstrated impressive accuracy in clinical decision-making, their performance declines when applied to unstructured, real-world clinical data rather than carefully curated datasets (53). Similarly, while Bayesian models offer consistency and transparency in reasoning, they are constrained by their dependence on predefined probabilities and structured inputs, features that may not reflect the ambiguity and nuance of clinical encounters. These limitations underscore the ongoing need for human oversight. Although hybrid systems that integrate probabilistic and generative models may enhance AI performance in the future, current applications of LLMs alone remain problematic. As such, medical education must prioritise the training of clinicians to critically appraise AI-generated recommendations, cultivate metacognitive awareness and ensure preparedness for contingency in case of AI failure (165). Such training is essential to mitigate the risks of overreliance and inappropriate application of AI in clinical practice. Finally, considerable work is needed to identify how AI directed CDSS systems can be optimally embedded into existing clinical workflows, where they work seamlessly alongside clinical personnel across a range of procedures (166). and whether they perform better than existing decision-support tools, practices, and diagnostic frameworks (167, 168).

## Conclusion

Clinical reasoning principally utilises three main strategies—rapid, intuitive pattern recognition and the slower, explicit, and more structured forms—scheme-inductive reasoning and hypothetico-deductive testing. Each of these is vulnerable to working-memory limitations, temporal decay, schema inefficiencies and cognitive bias. AI largely has the capability to mitigate these vulnerabilities by externalising complex data into interactive dashboards and saliency overlays, serving as an external schema repository incorporating real-time Bayesian updates and offering transparent, chain-of-thought explanations and metacognitive prompts. When integrated within the diagnostic workflow, it is able to contribute in refining causal models, guiding investigations, interpreting results and signalling premature closure. AI-driven clinical decision support would be able to decrease cognitive load, sharpen clinical judgement and permit the clinician to engage in deeper learning and metacognitive adjustment.

Thus, AI systems can provide a powerful complement to human reasoning. By fostering a synergistic partnership between clinicians and AI (169), the human cognitive limitations related to diagnostic reasoning can be addressed, allowing improved diagnostic accuracy and patient outcomes, where the combination of AI's data-driven insights and clinicians' contextual judgment ensures optimal care.
